# Significance of the Melanocortin 1 and Endothelin B Receptors in Melanocyte Homeostasis and Prevention of Sun-Induced Genotoxicity

**DOI:** 10.3389/fgene.2016.00146

**Published:** 2016-08-17

**Authors:** Viki B. Swope, Zalfa A. Abdel-Malek

**Affiliations:** Department of Dermatology, College of Medicine, University of Cincinnati, CincinnatiOH, USA

**Keywords:** melanocortin 1 receptor, endothelin B receptor, melanocytes, melanoma, ultraviolet radiation, photoprotection, DNA repair

## Abstract

The membrane bound melanocortin 1 receptor (MC1R), and the endothelin B receptor (ENDBR) are two G-protein coupled receptors that play important roles in constitutive regulation of melanocytes and their response to ultraviolet radiation (UVR), the main etiological factor for melanoma. The human MC1R is a G_s_ protein-coupled receptor, which is activated by its agonists α-melanocyte stimulating hormone (α-melanocortin; α-MSH) and adrenocorticotropic hormone (ACTH). The ENDBR is a G_q_ coupled-receptor, which is activated by Endothelin (ET)-3 during embryonic development, and ET-1 postnatally. Pigmentation and the DNA repair capacity are two major factors that determine the risk for melanoma. Activation of the MC1R by its agonists stimulates the synthesis of eumelanin, the dark brown photoprotective pigment. *In vitro* studies showed that α-MSH and ET-1 interact synergistically in the presence of basic fibroblast growth factor to stimulate human melanocyte proliferation and melanogenesis, and to inhibit UVR-induced apoptosis. An important function of the MC1R is reduction of oxidative stress and activation of DNA repair pathways. The human *MC1R* is highly polymorphic, and *MC1R* variants, particularly those that cause loss of function of the expressed receptor, are associated with increased melanoma risk independently of pigmentation. These variants compromise the DNA repair and antioxidant capacities of human melanocytes. Recently, activation of ENDBR by ET-1 was reported to reduce the induction and enhance the repair of UVR-induced DNA photoproducts. We conclude that α-MSH and ET-1 and their cognate receptors MC1R and ENDBR reduce the risk for melanoma by maintaining genomic stability of melanocytes via modulating the DNA damage response to solar UVR. Elucidating the response of melanocytes to UVR should improve our understanding of the process of melanomagenesis, and lead to effective melanoma chemoprevention, as well as therapeutic strategies.

## Introduction

**FIGURE 1:**
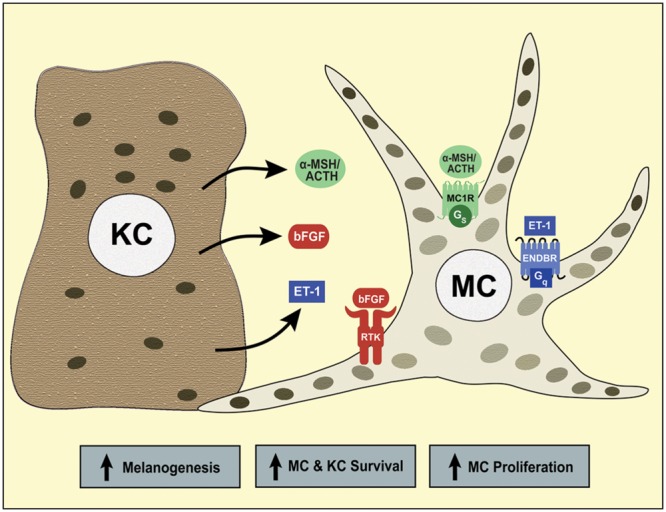
**Paracrine regulation of human melanocytes by the keratinocyte-derived melanocortins, ET-1 and basic fibroblast growth factor (bFGF).** Keratinocytes synthesize α-MSH and adrenocorticotropic hormone (ACTH), the agonists of MC1R, ET-1, the agonist of the endothelin B receptor (ENDBR), and bFGF, the ligand for bFGF receptor tyrosine kinase (RTK). When these receptors are activated, the crosstalk of their signaling pathways stimulates human melanocyte proliferation, melanogenesis and survival. Melanocytes transfer melanin-containing melanosomes to keratinocytes, a photoprotective mechanism that reduces the genotoxic effects of solar UVR.

Epidermal melanocytes are pigment producing cells that are derived from the neural crest during embryonic development and are responsible for the distinctive skin coloration (Adams and Bronner-Fraser, 2009). Once they populate the epidermis, the majority of melanocytes are fully differentiated, and similar to neurons, have a long life span and a low proliferative capacity. Disruption of the homeostasis of melanocytes results in pigmentary disorders, the most extreme of which are vitiligo, an acquired depigmentary disorder caused by loss of melanocytes, and melanoma, the deadliest form of skin cancer caused by uncontrolled proliferation due to mutations in genes such as *CDKN2A*, and/or by reduced DNA repair capacity due to mutations in genes such as the melanocortin 1 receptor gene (*MC1R*; Alikhan et al., 2011; Abbas et al., 2014). The significance of melanocytes lies in their photoprotective role against the genotoxic effects of solar ultraviolet radiation (UVR; Kadekaro et al., 2003). The pigment melanin, which is deposited in specialized organelles, melanosomes, that are transferred from melanocytes to keratinocytes, shields the nuclei of these cells from impinging UV rays, and also acts as a scavenger of reactive oxygen species that can damage DNA, proteins and lipids (Pathak et al., 1980; Bustamante et al., 1993). Genetic and *in vitro* studies showed that melanocyte homeostasis is regulated by many genes, including those that code for growth factor receptors, transcription factors, and their targets (Kondo and Hearing, 2011). *In vitro* studies showed that mammalian, including human, melanocytes are regulated by signaling pathways that are activated by G-protein coupled receptors and tyrosine kinase receptors (Halaban et al., 1988a; Abdel-Malek et al., 1995a,b; Swope et al., 1995; Imokawa et al., 1997; Kunisada et al., 1998; Tada et al., 1998; **Figure 1**). These pathways and their crosstalk regulate the melanocytes constitutively, as well as their response to their micro- and macro-environment, mainly solar UVR, the major etiological factor for melanoma.

### Role of Melanins in Photoprotection

Melanin produced by melanocytes, is the main photoprotective mechanism against UVR-induced photodamage, the underlying cause of skin cancers, including melanoma (Pathak et al., 1971; Pathak, 1995; Gilchrest et al., 1999). It is common knowledge that individuals with fair skin who have a poor tanning ability are at a higher risk for skin cancers, including melanoma, than individuals with dark skin (Dubin et al., 1989; Halder and Bridgeman-Shah, 1995). Human melanocytes synthesize both eumelanin, the dark brown pigment, and pheomelanin, the red–yellow pigment (Hunt et al., 1995a; Wakamatsu et al., 2006). The ratio of eumelanin to pheomelanin is an important determinant of skin color, and correlates directly with total melanin content. In human melanocytes, it is eumelanin, rather than pheomelanin content, that determines the extent of skin pigmentation (Hennessy et al., 2005; Wakamatsu et al., 2006). It is known that eumelanin is more photoprotective than pheomelanin due to its resistance to photodegradation, its ability to shield the skin from UVR and to scavenge reactive oxygen radicals (Menon et al., 1983; Nofsinger et al., 2002; Meredith and Sarna, 2006). Eumelanin content has been shown to correlate inversely with the induction of DNA photoproducts in the skin *in situ*, as well as in human melanocytes *in vitro* (Tadokoro et al., 2003; Hauser et al., 2006). Pheomelanin, on their other hand, is photolabile and can be a pro-oxidant (Panzella et al., 2014). It was reported that pheomelanin promotes melanomagenesis via induction of oxidative DNA damage in mice harboring the activating Braf^v600E^ mutation, without exposure to any carcinogen, such as UVR (Mitra et al., 2012). This suggests that pheomelanin can be oncogenic. Recently, the induction of UVR-induced DNA photoproducts in melanin-containing melanocytes and keratinocytes was found to increase even after cessation of UVR exposure, and this increase was greater in the skin of *K14-Kitl* / *mc1r^e/e^* (recessive yellow) mice than in the skin of black *K14-Kitl* mice expressing wild type *mc1r* (Premi et al., 2015). This difference in DNA photoproducts was attributed to pheomelanin in the skin of transgenic recessive yellow mice. The elevated DNA photoproducts in these mice can possibly overwhelm the DNA repair capacity, thus increasing the chance for mutations, the underlying cause of skin cancer, including melanoma. These findings clearly indicate that the photoprotective effects of eumelanin vs. pheomelanin are not merely due to their differential ability to act as a physical barrier that reduces the penetration of UVR through the epidermal layers, and emphasize the significance of elucidating the role of these melanins in determining the risk for melanoma.

## The MC1R, a G_s_ Protein-Coupled Receptor Expressed on Melanocytes

The main regulator of eumelanin synthesis in mammalian melanocytes is the melanocortin 1 receptor (MC1R), a G_s_ protein-coupled receptor that is activated upon binding of α-melanocyte stimulating hormone (α-melanocortin; α-MSH; Silvers, 1961; Mountjoy et al., 1992). Genetic studies on mouse coat color identified the *mc1r* as the major regulator of pheomelanin/eumelanin switch in follicular melanocytes (Geschwind et al., 1972; Tamate and Takeuchi, 1984). The *recessive yellow* mutation in mouse *mc1r* results in loss of function (LOF) of the receptor and in yellow coat color, due to the exclusive production of pheomelanin (Tamate and Takeuchi, 1984; Robbins et al., 1993). *In vivo* studies showed that injection of human subjects with purified melanocortins induced an increase in pigmentation (Lerner and McGuire, 1961). This effect was corroborated by the finding that injection of human subjects with the potent α-MSH analog NDP-MSH increased skin pigmentation in the absence of any sun exposure (Levine et al., 1991). However, from these studies, it could not be determined whether or not melanocortins had a direct effect on melanocytes. In the early 1990’s, the cloning of the *MC1R* from human melanocytes and the demonstration that the expressed receptor is functional provided strong evidence in support of the significance of MC1R in regulating human pigmentation by directly affecting melanin synthesis in melanocytes (Mountjoy et al., 1992; Abdel-Malek et al., 1995b). As its mouse ortholog, activation of the human MC1R by the potent α-MSH analog NDP-MSH increased the synthesis of eumelanin by human melanocytes (Hunt et al., 1995b). It was established that α-MSH and ACTH bind the human MC1R with the same affinity, thus both are considered to be physiological agonists of the receptor (Suzuki et al., 1996). As has been shown for the mouse mc1r, agouti signaling protein functions as an inverse agonist of the human MC1R, competitively inhibiting the binding of α-MSH and reducing the basal levels of tyrosinase, the rate-limiting enzyme in the melanin synthetic pathway (Suzuki et al., 1997). More recently, it was demonstrated that human β-defensin 3 also functions as an antagonist of the human MC1R (Candille et al., 2007; Swope et al., 2012).

### The cAMP Pathway, the Main Signaling Pathway Activated by MC1R

The MC1R is a G_s_ protein-coupled receptor with seven transmembrane domains that is expressed on the cell surface of melanocytes and signals mainly through activation of the cAMP pathway (Suzuki et al., 1996; Garcia-Borron et al., 2014). It belongs to the family of melanocortin receptors (MC1-5R), and is the only MCR expressed on melanocytes (Suzuki et al., 1996). Since the 1970’s, it has been known that α-MSH activates the synthesis of cAMP, as demonstrated *in vitro* using mouse melanoma cells, and that the rise in cAMP levels stimulates the activity of tyrosinase, thus increasing melanin synthesis (Kreider et al., 1973; Pawelek et al., 1973; Wade and Burkart, 1978; Legros et al., 1981). The effect of α-MSH on pigmentation was mimicked by dibutyryl cAMP (db cAMP), and by phosphodiesterase inhibitors, such as theophylline and isobutyl methylxanthine (IBMX), that prevent cAMP degradation. Injection of newborn mice with either α-MSH or db cAMP resulted in increased number of dopa-positive melanocytes, suggesting stimulation of melanoblast differentiation (Hirobe and Takeuchi, 1977). These early studies established that α-MSH activates the cAMP pathway, which mediates its pigmentary effect.

Later, the cAMP pathway was reported to enhance the proliferation and stimulate pigmentation of primary mouse and human melanocytes (Eisinger and Marko, 1982; Tamura et al., 1987; Abdel-Malek et al., 1992). Treatment of human melanocytes with α-MSH increases the activity of tyrosinase, and this effect is mimicked by forskolin, a direct activator of adenylate cyclase (Abdel-Malek et al., 1995b; Scott et al., 2002; Kadekaro et al., 2010). The proliferation of human melanocytes in culture is stimulated by the crosstalk of the cAMP-, protein kinase C (PKC)- and/or tyrosine kinase receptors-dependent pathways. The first culture medium for primary human melanocytes contained 12-*o*- tetradecanoylphorbol ester (TPA), which activates PKC, and cholera toxin and IBMX, which increase intracellular cAMP levels by activating adenylate cyclase and inhibiting phosphodiesterase, respectively (Eisinger and Marko, 1982). By binding and activating their cognate receptor tyrosine kinases, basic fibroblast growth factor (bFGF), hepatocyte growth factor (HGF) and stem cell factor (SCF) stimulate melanocyte proliferation in the presence of a cAMP stimulator (Halaban et al., 1988b; Matsumoto et al., 1991; Grichnik et al., 1998). Human melanocyte cultures can be established and maintained in growth medium containing bFGF, endothelin-1 (ET-1), which increases intracellular calcium concentration and activates PKC, and α-MSH (Swope et al., 1995). These three factors interact synergistically to stimulate melanocyte proliferation and melanogenesis (**Figure 1**). The crosstalk of the signaling pathways of these growth factors activates the MAP kinases ERK1/2, known to regulate melanocyte proliferation (Bohm et al., 1995; Tada et al., 1998). Interestingly, bFGF, SCF, HGF, ET-1, α-MSH, and ACTH are all synthesized in the epidermis, thus function as paracrine factors that maintain melanocyte homeostasis (Halaban et al., 1988a; Imokawa et al., 1992; Schauer et al., 1994; Wakamatsu et al., 1997; Kakurai et al., 2002; Mildner et al., 2007).

### MC1R Variants and Their Impact on Receptor Function

The *MC1R* is a major contributor to the diversity of human pigmentation. Epidemiological studies identified many polymorphisms of the *MC1R* (close to 200 allelic variants) that are expressed in different human populations (Garcia-Borron et al., 2014). The wild type *MC1R* is predominantly expressed in Africa, where dark skin with high eumelanin content is needed for adaption to the equatorial sun (Harding et al., 2000). Certain variants associated with fair skin and red hair phenotype, mainly R151C, R160W, and D294H, are highly expressed in Northern European populations, and in the Celtic population of Australia (Box et al., 1997; Smith et al., 1998). The association of this pigmentary phenotype with increased melanoma risk implicated the *MC1R* in melanoma predisposition (Palmer et al., 2000; Box et al., 2001; Kennedy et al., 2001; van der Velden et al., 2001; Landi et al., 2005; Kanetsky et al., 2006). However, expression of the above three allelic variants is necessary but not sufficient for red hair phenotype, as they are also expressed in individuals with dark skin and hair color (Palmer et al., 2000; Kennedy et al., 2001; Landi et al., 2005; Han et al., 2006; Stratigos et al., 2006; Pasquali et al., 2015). Expression of these variants increased melanoma risk independently of skin or hair color, as confirmed by studies conducted on Southern Italian and Greek populations. Analysis of large data that included 5160 melanoma cases and 12,119 controls revealed that expression of the nine most common *MC1R* variants, R151C, R160W, D294H, V60L, R142H, D84E, I155T, R163Q, and V92M, increased melanoma risk, and this was observed in darkly pigmented Caucasians (Pasquali et al., 2015). These findings confirm previous results showing that carriage of two low risk *MC1R* variants (V92M or R163Q), or any high risk variant (R151C, R160W, or D294H) is associated with increased melanoma risk, mostly in individuals with dark skin, limited sun exposure, and good tanning response (Kanetsky et al., 2010). These epidemiological results concluded that melanoma risk cannot be accurately ascertained based only on pigmentary phenotype, and suggested that MC1R regulates other functions in melanocytes, independently of pigmentation.

Using primary cultures of human melanocytes expressing different *MC1R* genotypes, or heterologous cells transfected with wild type or a *MC1R* variant, revealed that the red hair allelic variants, R151C, R160W, and D294H, result in LOF of the MC1R by impairing its functional coupling to G_s_ protein, thus inhibiting its signaling via increasing cAMP synthesis (Scott et al., 2002; Ringholm et al., 2004; Kadekaro et al., 2010). Others reported that melanocytes homozygous for R151C or R160W have impaired binding of α-MSH to the MC1R (Beaumont et al., 2005). However, this was not the case for D294H variant. Expression of any two of these three variants as homozygous or compound heterozygous causes loss of MC1R function in human melanocytes, as determined by lack of response to α-MSH by increasing intracellular cAMP levels and tyrosinase activity (Scott et al., 2002; Kadekaro et al., 2010). Melanocytes expressing LOF MC1R responded normally to forskolin, confirming that the inability to respond to α-MSH is due to a defect at the level of the receptor, and not its downstream signaling pathway. The variant V60L, which is associated with blond hair, results in reduction, not total loss, of MC1R activity (Kadekaro et al., 2010). The very common alleles V92M, and R163Q that is mainly expressed in Southeast Asia, did not alter the activity of the MC1R, and are considered as pseudoalleles. Despite the findings that the activity of the MC1R encoded for by R163Q variant is comparable to that encoded by the wild type allele, the resulting pigmentary phenotypes are not identical, suggesting that allelic variants of the *MC1R* alter the interaction of MC1R with other genes that regulate pigmentation.

The MC1R has seven transmembrane domains. Site-directed mutagenesis of the MC1R revealed that the second transmembrane domain is critical for agonist binding, and the second intracellular loop is essential for coupling to G_s_ and stimulating cAMP synthesis (Sanchez Mas et al., 2002). The R151C and R142H substitutions, which are associated with red hair and result in impaired MC1R signaling, are both located in the second intracellular loop of the receptor. Some *MC1R* variants impair the trafficking of the receptor and hence reduce its cell surface expression. This has been reported for the red hair allelic variants R151C and R160W, as well as for D84E and I155T, but not for the red hair variant D294H (Beaumont et al., 2005). Expression of *MC1R* variants had no impact on MC1R protein levels, suggesting that reduced number of membrane bound receptors is not due to decreased stability of the protein. It was reported that R151C impairs MC1R trafficking by retaining it in the endoplasmic reticulum, and that R160W results in MC1R retention in the *cis*-Golgi (Sanchez-Laorden et al., 2009). Phosphorylation of T157 and the ^160^RARR^163^ motif were shown to be critical for normal receptor trafficking and are conserved in all five members of the melanocortin receptors family.

### Effect of MC1R on UVR-Induced DNA Damage in Melanocytes

Given the significance of pigmentation in photoprotection and that tanning is a hallmark of exposure to UVR, the role of melanocortins and the MC1R in the response of human melanocytes to UVR has been the subject of investigation in a number of laboratories. It was reported that activation of the cAMP pathway is critical for stimulating melanogenesis in cultured human melanocytes exposed to UVR (Im et al., 1998). Later, it was reported that melanocytes expressing two red hair allelic variants, hence LOF MC1R, have increased sensitivity to UVR, as determined by increased melanocyte death by apoptosis following UVR exposure (Scott et al., 2002). During the past decade, a novel and paradigm shifting role for MC1R signaling was described, namely enhancement of repair of UVR-induced DNA damage in human melanocytes. The ability of α-MSH to enhance repair of UVR-induced DNA photoproducts was first reported independently by us and by Bohm et al. (2005; Kadekaro et al., 2005). It is known that DNA photoproducts are formed by the direct absorption of UVR by pyrimidine bases in DNA, predominantly at sites containing a thymine (TC, TT; Budden and Bowden, 2013). These photoproducts create bulky lesions that distort the DNA helix, forming adducts that can halt transcription and DNA replication. If not repaired efficiently, the sustained DNA damage due to presence of photoproducts can result in mutations, the underlying cause of skin cancers, including melanoma. The significance of the activated MC1R and the cAMP pathway on DNA repair was further confirmed by other investigators (Smith et al., 2008; Jarrett et al., 2014). These reports established the role of the MC1R in regulating DNA repair in melanocytes, in addition to its well- known function as the main regulator of eumelanin synthesis.

Irradiation of melanocytes with UVR also results in the generation of oxidative DNA damage, in addition to DNA photoproducts (Denat et al., 2014). We showed that treatment of melanocytes with α-MSH reduced the UVR-induced generation of reactive oxygen species, increased the activity and levels of catalase, a first-line-of-defense antioxidant enzyme, as well as the levels of ferritin, another anti-oxidant protein (Song et al., 2009). As expected, these effects resulted in reduced generation of 8-oxodeoxyguanosine (8-oxodG), the major form of oxidative DNA damage. Others reported the effects of α-MSH on activation of Nrf2, a transcription factor that regulates the expression of genes that code for phase II detoxifying enzymes, such as heme oxygenase-1 and glutathione *s*-transferase Pi, by binding to the antioxidant response elements in their promoters (Kokot et al., 2009). Another transcription factor that was found to be pivotal for reducing oxidative DNA damage in melanocytes is p53 (Kadekaro et al., 2012). Treatment with α-MSH contributed to the UVR-induced accumulation of p53 in melanocytes, and silencing of p53 abolished the inhibitory effect of α-MSH on hydrogen peroxide-induced oxidative DNA damage. Activation of transcription factors, such as Nrf2 and p53, by α-MSH strongly suggested its transcriptional regulation of antioxidant genes involved in the response of melanocytes to UVR.

Microarray experiments provided compelling evidence for the transcriptional effects of MC1R, particularly on genes that are involved in the regulation of the cell cycle, DNA repair, antioxidant defenses, apoptosis, and pigmentation (Kadekaro et al., 2010). Treatment with α-MSH reversed the effects of UVR on the expression of many genes involved in these functional categories, which is consistent with the results of functional assays demonstrating reduced oxidative DNA damage and enhanced repair of DNA photoproducts and survival. Importantly, we reported that these effects of α-MSH require expression of functional MC1R, and are absent in melanocytes expressing LOF MC1R, due to expression of two red hair color allelic variants. The findings that melanocytes with LOF MC1R have reduced DNA repair and antioxidant capacities provide an explanation for the vulnerability of these melanocytes to malignant transformation to melanoma.

#### MC1R and the DNA Damage Response of Melanocytes to UVR

**FIGURE 2:**
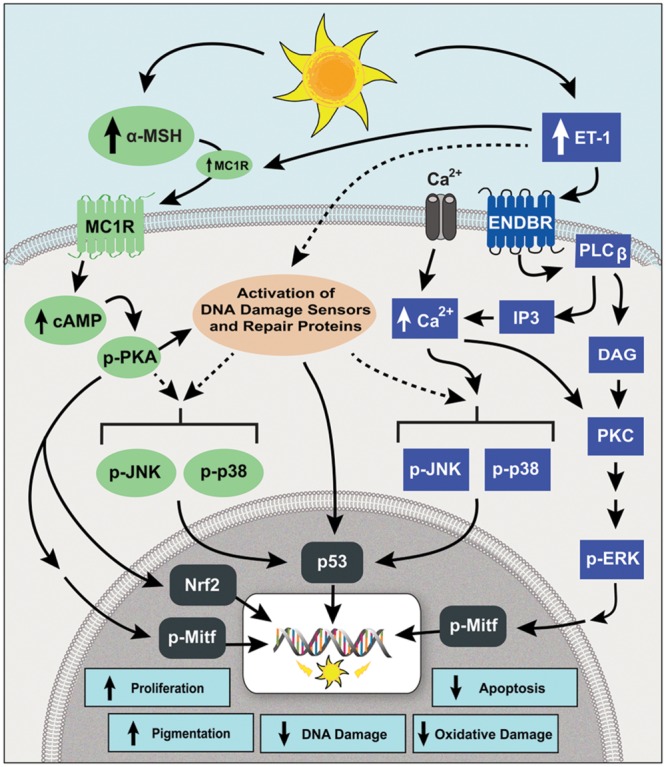
**The signaling pathways of MC1R and ENDBR modulate the DNA damage response (DDR) of human melanocytes to UVR.** Irradiation with UVR increases the synthesis and release of α-MSH and ET-1 by keratinocytes. Binding of α-MSH to MC1R stimulates the synthesis of cAMP that activates PKA, which in turn activates DNA damage sensors, increases DNA repair proteins, and activates the transcription factor Nrf-2, which increases the expression of phase II antioxidant genes. Endothelin-1 binds ENDBR and activates intracellular Ca^2+^ mobilization and PKC, leading to phosphorylation of ERK1/2, and enhances the UVR-induced phosphorylation of JNK and p38. The signaling pathways of MC1R and ENDBR converge on activation of the transcription factors MITF and p53, resulting in increased melanocyte survival, repair of UVR-induced DNA damage, antioxidant defenses and pigmentation.

Exposure to UVR triggers a DNA damage response (DDR), a signal transduction pathway that coordinates cell cycle transition, DNA repair, and apoptosis, in order to prevent genomic instability (Cimprich and Cortez, 2008). Efficient DNA repair, an integral component of the DDR to UVR, is critical for evasion of photocarcinogenesis (Kraemer et al., 1994; DiGiovanna and Kraemer, 2012). The role of MC1R in the DDR of human melanocytes to UVR is illustrated in **Figure 2**. Our investigation of the role of the MC1R in the DDR of melanocytes to UVR revealed that activation of the MC1R by α-MSH binding resulted in the phosphorylation, hence activation of the DNA damage sensors ataxia telangiectasia mutated (ATM) and Rad3 related (ATR), ATM, and DNA-PK (Kadekaro et al., 2012; Swope et al., 2014). Treatment with α-MSH increased the levels of Chk1 and Chk2, the immediate downstream targets of ATR and ATM, as well as the transcription factor p53, and γ-H2AX, the phosphorylated form of histone 2AX (Swope et al., 2014). Accumulation of p53 and its transactivation are important for cell cycle arrest, DNA repair, and apoptosis of cells with damaged DNA (Gomez-Lazaro et al., 2004). Formation of γ-H2AX is critical for recruitment of DNA repair proteins to DNA damage sites (Celeste et al., 2002). As expected, these effects of α-MSH were absent in melanocytes expressing LOF MC1R. Treatment with α-MSH also increased the levels of XPC, the enzyme involved in recognition of DNA damage, the first step in nucleotide excision repair, the main repair pathway for DNA photoproducts (Hanawalt, 2002). The significance of activation of DNA damage sensors by α-MSH was further demonstrated by the findings that activation of the MC1R, which results in increased cAMP levels and activation of the cAMP-dependent PKA, induces the phosphorylation of ATR on Ser 435, and that phosphorylation of this site is required for association of ATR with the DNA repair protein XPA (Jarrett et al., 2014). Collectively these results provide compelling evidence for the significance of the MC1R and its signaling pathway in modulating the response of melanocytes to UVR, which is expected to maintain genomic stability and prevent melanoma formation.

## The Endothelin-B Receptor, a G_q_ Protein-Coupled Receptor Expressed on the Cell Surface of Melanocytes

Another important G-protein coupled receptor with critical role in the maintenance of melanocyte survival and homeostasis is the Endothelin-B receptor (ENDBR), a G_q_-coupled receptor that is expressed on the cell surface of melanocytes (Tada et al., 1998; Mizuno et al., 2005). The ENDBR is the receptor for Endothelin (ET)-3 that is expressed during prenatal life on melanoblasts, the precursors of melanocytes (Lee et al., 2003). Activation of ENDBR by ET-3 binding is critical for the survival and migration of neural crest-derived cells, including melanoblasts. Mutations that disrupt the expression of either *ENDBR* or *ET-3* gene result in Hirschsprung’s disease, characterized by unpigmented skin lesions due to loss of melanoblasts, and a megacolon due to absence of enteric ganglia that have the same embryonic origin as melanocytes (Baynash et al., 1994). The ENDBR is also the receptor of ET-1 that is expressed postnatally, and has the same binding affinity and effects as ET-3 (Tada et al., 1998). There are two ET receptors, ENDAR and ENDBR (Rubanyi and Polokoff, 1994). The latter is the predominant receptor expressed by human melanocytes (Tada et al., 1998). Activation of ENDBR by ET-1 increases intracellular Ca^2+^ mobilization and PKC activity. Endothelin-1 is a 21-amino acid peptide that is derived from a larger inactive pro-peptide (Rubanyi and Polokoff, 1994). It is synthesized by keratinocytes, and its expression is increased upon UVR exposure (Imokawa et al., 1992, 1995).

### Role of ET-1/ENDBR Axis in the Regulation of Melanocytes and the Crosstalk of ENDBR and MC1R Signaling Pathways

Endothelin-1 was first described as a potent mitogen for human melanocytes (Yada et al., 1991; Imokawa et al., 1992). We reported that ET-1 interacts synergistically with α-MSH and bFGF to stimulate human melanocyte proliferation and melanogenesis, and to reduce UVR-induced apoptosis (Swope et al., 1995; Tada et al., 1998; Kadekaro et al., 2005). The synergistic effects of ET-1, α-MSH and bFGF led us to utilize them in a growth medium for maintenance of human melanocytes in culture, in place of non-physiological mitogens, such as TPA or cholera toxin (Swope et al., 1995). Activation of ENDBR also reduces the generation of reactive oxygen species, which should decrease oxidative DNA damage (Kadekaro et al., 2005). The signaling pathways and the effects of ET-1 are summarized in **Figure 2**. Activation of ENDBR results in increased phosphorylation of the mitogen activated MAP kinase ERK1/2, the transcription factor CREB, and the downstream transcription factor Mitf (Tada et al., 2002; Kadekaro et al., 2005). The synergistic interaction of ENDBR with MC1R involves enhanced activation of Mitf, increased expression of its target the anti-apoptotic Bcl2, and increased phosphorylation of Akt, which increases survival by inhibiting the pro-apoptotic Bad (Kadekaro et al., 2005). We also reported that treatment of human melanocytes with ET-1 increases their migration, while treatment with α-MSH promotes adhesion (Scott et al., 1997). Another important effect of ET-1 is up regulation of expression of *MC1R*, which is expected to increase MC1R expression on the cell surface of melanocytes, and sustain their response to α-MSH (Tada et al., 1998; Swope et al., 2012). These results demonstrate the significance of ENDBR and MC1R, two distinct G protein-coupled receptors, and the various means of interaction of their signaling pathways in regulating melanocyte survival and function. Additionally, these results underscore the significance of the paracrine factors α-MSH and ACTH, and ET-1, in maintenance of melanocyte homeostasis.

### Effect of ENDBR/ET-1 Axis in Repair of UV-Induced DNA Photoproducts

Recently, we found that treatment with ET-1 reduces the induction and enhances the repair of cyclobutane pyrimidine dimers (CPDs), the major form of DNA photoproducts, in UVR-irradiated human melanocytes (von Koschembahr et al., 2015). The effects of ENDBR/ET-1 axis on the DDR response of human melanocytes to UVR are summarized in **Figure 2**. Endothelin-1 was more effective than α-MSH in enhancing CPD repair, since this effect was evident at concentrations ≤ 1 nM, compared to 10 nM α-MSH. The effects of ET-1 on CPD repair and survival were mediated by increased intracellular Ca^2+^ mobilization, and the downstream activation of the stress-induced MAP kinases JNK and p38. Others have reported that increased expression of ET-1 reduced UVR-induced CPDs in mouse skin (Hyter et al., 2013). An important finding is that treatment with ET-1 enhanced repair of CPDs in melanocytes expressing LOF MC1R, suggesting that activation of ENDBR and its signaling pathways can compensate for loss of MC1R signaling in melanocytes (von Koschembahr et al., 2015). Therefore activation of ENDBR is a mechanism to counteract the genotoxic effects of UVR in melanocytes, regardless of their *MC1R* genotype. We are further investigating the effects of ENDBR and MC1R signaling on the DDR of melanocytes to UVR to identify common, as well as distinct downstream effectors that are involved in reducing the genotoxic effects of UVR.

## Melanocortin Analogs for Prevention of Skin Cancers, Including Melanoma

For decades, there has been interest in developing melanocortin analogs that can stimulate pigmentation without sun exposure (sunless tanning). There were also many attempts to tag melanocortin analogs to chemotherapeutic agents to selectively eradicate melanoma tumor cells (Abdel-Malek, 2010). The best known and most studied melanocortin analog is NDP-MSH (Ac-[Nle^4^, D-Phe^7^]-α-MSH), which proved to be at least 100-fold more potent, and markedly most stable and with more prolonged effects than the native α-MSH in amphibians, reptiles and mammals (Sawyer et al., 1980, 1982; Marwan et al., 1985). NDP-MSH was also more potent and had a more prolonged effect than α-MSH on tyrosinase activity of cultured human melanocytes (Abdel-Malek et al., 2006). Systemic administration of NDP-MSH to human volunteers demonstrated its efficacy in inducing pigmentation in the absence of any sun exposure (Levine et al., 1991). More recently, the photoprotective effect of NDP-MSH was demonstrated by the reduction of sun-induced DNA damage in subjects treated with this analog (Barnetson et al., 2006). However, the administration of NDP-MSH resulted in side effects, including nausea and loss of appetite (Levine et al., 1991), which can be attributed to binding and activating other melanocortin receptors expressed on cells other than melanocytes.

With the discovery that α-MSH enhances DNA repair and activates antioxidant defenses, in addition to stimulating eumelanin synthesis, we became interested in developing small analogs of α-MSH with high selectivity and specificity for the MC1R. This is important in drug development in order to avoid any off-target effects. It has been shown that the core 6–9 amino acid residues of α-MSH, His-Phe-Arg-Trp, are required for its melanogenic activity (Hruby et al., 1987; Castrucci et al., 1989). Based on this, we developed *n*-capped tetrapeptide analogs, containing this core sequence, or tripeptides, consisting of His-Phe-Arg sequence, in which L-Phe was replaced by D-Phe, which is known to confer stability to the peptide (Abdel-Malek et al., 2006; Abdel-Malek et al., 2009). These *n*-capped analogs were tested for their potency on human melanocytes, using stimulation of cAMP synthesis and tyrosinase activity as two endpoints. Based on these assays, we selected the most potent tetra- and tripeptide analogs, some of which proved to be highly selective for MC1R, with very low, or no affinity for the remaining melanocortin receptors. These analogs, like α-MSH, enhanced repair of CPDs and reduced the generation of reactive oxygen species in UVR-irradiated human melanocytes, and are being further tested in preclinical assays to insure their efficacy and safety. The lipophilic property of these analogs, together with their small size and low molecular weight, is expected to enhance their skin permeation when applied topically. Similar to α-MSH, these analogs require the expression of functional MC1R, and will have no effect on individuals expressing LOF MC1R. Despite this limitation, developing these analogs as a skin cancer, including melanoma, prevention strategy will benefit millions who are heterozygous for *MC1R* variants, and have a high risk for melanoma due to reduced MC1R activity. Carriers of one *MC1R* variant represent about 50% of all White Caucasians (Schaffer and Bolognia, 2001). Our analogs should also benefit individuals who are carriers of mutations in other melanoma predisposition genes, such as *CDKN2A*.

Our finding that activation of ENDBR enhances repair of DNA photoproducts in melanocytes makes it attractive to harness the ENDBR signaling pathway for a melanoma prevention strategy that can globally benefit high risk individuals, regardless of *MC1R* genotype (von Koschembahr et al., 2015). The ENDBR is ubiquitously expressed, thus it cannot be targeted directly for melanoma prevention. However, downstream effectors of ENDBR that impact the DDR of melanocytes can potentially be targeted for this purpose. Studies are underway to identify such downstream effectors that can also be activated by MC1R, or are specific to ENDBR signaling.

## Conclusion

The MC1R and ENDBR are two important G protein-coupled receptors that have multiple effects on melanocytes, ensuring their homeostasis, and modulating their DDR to UVR by reducing DNA damage, enhancing survival, and stimulating melanin synthesis. The human MC1R agonists, α-MSH and ACTH, and ENDBR ligand, ET-1, are paracrine factors, whose synthesis by epidermal keratinocytes is increased upon UVR exposure. These ligands, in turn, up regulate the expression of the *MC1R.* Additionally, α-MSH and by analogy ACTH, interact synergistically with ET-1 to stimulate melanocyte proliferation and survival. The vast interest in *MC1R* stems from its high polymorphism and its role as a melanoma susceptibility gene. The novel finding that ET-1, similar to α-MSH and ACTH, enhances DNA repair in melanocytes, identifies a novel mechanism by which melanocytes evade UVR-induced photocarcinogenesis. That activation of ENDBR signaling can compensate for LOF of MC1R opens the door for designing novel prevention strategies for high risk individuals, irrespective of their *MC1R* genotype.

## Author Contributions

Both authors made substantial intellectual contribution to the review and approved it for publication.

## Conflict of Interest Statement

The authors declare that the research was conducted in the absence of any commercial or financial relationships that could be construed as a potential conflict of interest.
